# Comparative Evaluation of qPCR and Digital PCR for the Detection of *Nosema ceranae* in Honey Bees

**DOI:** 10.3390/vetsci12121175

**Published:** 2025-12-10

**Authors:** Cecilia Guasco, Paola Mogliotti, Roberto Zoccola, Maria Goria, Alessandro Gamberoni, Paola Ghisellini, Patrizia Garbati, Cristina Rando, Stefano Ottoboni, Raffaella Barbieri, Roberto Eggenhöffner

**Affiliations:** 1 Istituto Zooprofilattico Sperimentale del Piemonte, Liguria e Valle d’Aosta, Via Bologna 148, 10154 Torino, Italy; cecilia.guasco@izsplv.it (C.G.); paola.mogliotti@izsplv.it (P.M.); roberto.zoccola@izsplv.it (R.Z.); maria.goria@izsplv.it (M.G.);; 2 Department of Surgical Sciences and Integrated Diagnostics (DISC), University of Genova, Corso Europa 30, 16132 Genova, Italy; paola.ghisellini@unige.it (P.G.); cristina.rando@unige.it (C.R.); stefano.ottoboni@unige.it (S.O.); 3 Istituto di Biofisica, Consiglio Nazionale delle Ricerche, 16149 Genova, Italy; patrizia.garbati@ibf.cnr.it (P.G.); raffaella.barbieri@ibf.cnr.it (R.B.)

**Keywords:** *Nosema ceranae*, honey bee health, molecular diagnostics, apiculture

## Abstract

*Apis mellifera* honey bees are keystone species for agriculture and global biodiversity, yet their populations are increasingly affected by multiple stressors, including the microsporidian parasite *Nosema ceranae*. Early and accurate detection of this pathogen is critical to prevent colony losses and sustain pollination services. This study compares qPCR and ddPCR for detecting *N. ceranae* in bees and hive debris. qPCR is suitable for routine screening, whereas ddPCR offers higher sensitivity and precise quantification. The high diagnostic concordance between the two assays in bee samples, coupled with ddPCR’s enhanced detection capability in debris, underscores their complementary value in apicultural monitoring. This study provides the first experimental comparison between qPCR and ddPCR applied to matched field samples of bees and hive debris, establishing a sensitive and practical diagnostic framework for *Nosema ceranae* surveillance. The findings support improved diagnostic accuracy and early detection in apicultural health programs.

## Simple Summary

Honey bees (*Apis mellifera*) are essential pollinators in both agricultural and natural ecosystems, but their populations are increasingly threatened by multiple stress factors, including pathogens. Among these, the microsporidian parasite *Nosema ceranae* is one of the most prevalent and harmful. This intracellular parasite shortens the lifespan of infected bees and often spreads asymptomatically, making early and accurate detection essential for effective disease prevention and colony protection. This study evaluates two molecular methods for detecting *Nosema ceranae* in honey bee samples. The results demonstrate the complementary strengths of these techniques: qPCR is suitable for routine surveillance, while ddPCR offers higher sensitivity and precise quantification, particularly in cases of low-level or environmental infections. By enhancing diagnostic accuracy and sensitivity, these methods enable beekeepers, veterinarians, and diagnostic laboratories to detect infections earlier, implement targeted control measures, and ultimately promote the health and sustainability of pollinator populations.

## 1. Introduction

Honey bees (*Apis mellifera*) are essential pollinators that support both agricultural and natural ecosystems, playing a vital role in global food security and biodiversity [1,2,3]. However, in recent decades, widespread colony losses have been reported by beekeepers driven by a complex interplay of biological, environmental, and anthropogenic stressors [4,5,6]. Among these stressors, pathogens—particularly microsporidia of the *Nosema*/*Vairimorpha* group—have emerged as significant threats to honey bee health due to their persistence, global distribution, and economic impact [7,8,9,10]. Although *N. ceranae* has been reclassified under *Vairimorpha* [11], we use the conventional name for diagnostic clarity [12]. *N. ceranae* has become the dominant microsporidian species infecting *A. mellifera* populations across Europe and other temperate regions, progressively displacing *N. apis* [13,14,15,16]. The parasite invades the epithelial cells of the bee’s midgut [17], disrupting nutrient absorption and metabolism. These disruptions lead to energetic stress, reduced foraging performance, and shortened lifespan [18,19,20]. At the colony level, chronic infections are associated with decreased productivity and increased susceptibility to other pathogens [10,21,22]. Since *N. ceranae* can severely damage bees, an early and accurate diagnosis is essential in order to assess the colony’s health status and avoid productivity losses [23].

Microscopic examination of spores has long served as the standard diagnostic method for Nosema infections due to its accessibility, low cost, and rapid execution [24,25]. Microscopy remains valuable as a preliminary tool, especially in high-intensity infections, where the characteristic oval spores are easily identified under 200–400× magnification. However, its limitations are well recognized: microscopy cannot distinguish between *N. apis* and *N. ceranae*, and it may also fail to detect early-stage or low-intensity infections [4,26,27]. For this reason, molecular diagnostics have become indispensable for reliable species-level identification and quantification.

The Polymerase Chain Reaction (PCR) assays targeting conserved genomic regions—such as the small subunit ribosomal RNA (SSU rRNA) and the heat-shock protein 70 (Hsp70) genes—enable species-specific identification [15,28]. Quantitative PCR (qPCR) represents a major improvement by allowing semi-quantitative detection based on real-time fluorescence and threshold cycle (Ct) analysis [29,30,31,32]. When properly standardized, qPCR provides sensitivity, reproducibility, and throughput suitable for large-scale surveillance. Nevertheless, its performance can be compromised by amplification inhibitors or stochastic variation near the detection limit, particularly when analyzing complex matrices such as hive debris [33,34,35].

Droplet digital PCR (ddPCR) represents a further advancement, providing absolute quantification of DNA targets without the need for calibration curves [36,37]. By partitioning reactions into thousands of nanoliter droplets and analyzing amplification events independently, ddPCR minimizes the impact of inhibitors and enhances detection sensitivity in low-copy samples [38,39]. The method is efficient and sensitive for pathogen quantification in veterinary and environmental microbiology [40,41]. However, despite its potential, ddPCR has not been systematically applied to the detection of *N. ceranae* in honey bees or environmental matrices.

Hive debris, a heterogeneous material that accumulates naturally at the bottom of hives, provides a non-invasive sampling matrix that integrates colony-level information over time [42]. Because debris contains traces of spores, wax, feces, and bee fragments, it reflects infection dynamics and can serve as an early indicator of colony health [10]. The capacity of ddPCR to detect *N. ceranae* DNA in debris with high sensitivity could thus represent a significant step forward in apicultural diagnostics, complementing microscopy and qPCR-based screening.

This study compares qPCR and ddPCR for detecting *N. ceranae*, evaluating their sensitivity, accuracy, and practical application in bee health monitoring.

## 2. Materials and Methods

### 2.1. Study Location and Sampling

Sampling was conducted at three apiaries in the Asti province of the Piedmont region (see Table 1). Samples were collected during spring and summer 2022, the periods typically associated with higher *N. ceranae* activity [42]. In each apiary, at least two hives were randomly selected to provide colony-level replication and to minimize site bias.

For each colony, two types of samples were collected: foraging bees and hive debris. Each bee sample consisted of a pool of at least 30 foraging workers collected at the hive entrance using sterile forceps. Hive debris (approximately 50 mg per colony) was collected from multiple points across the hive floor to obtain a representative composite sample and to reduce spatial variability within the colony environment. All samples were placed in sterile 1.5 mL microtubes, transported in insulated containers to prevent thermal degradation, and stored at −20 °C immediately upon arrival at the laboratory.

This approach balances reliable molecular detection with minimal colony disturbance. Although the number of individuals or debris mass can vary slightly among field studies, our design follows well-established protocols commonly adopted for the diagnosis of *N. ceranae* infections in managed honey bee colonies [40]. Pooling foragers in groups of 30 provides a representative estimate of colony-level infection, while debris samples of 50 mg are convenient for manipulation without loss of diagnostic sensitivity. This approach, refined through our prior field and diagnostic experience, balances the need for analytical accuracy with the operational constraints of apiary work.

### 2.2. Sample Preparation

Bee and debris samples were prepared under sterile conditions using standardized protocols to maximize reproducibility and DNA yield. Each bee was handled individually using sterile forceps, grasping it by the thorax and abdomen to minimize external contamination.

For molecular processing, intestines from ten worker bees were dissected and pooled in 1 mL of double-distilled water, and then subjected to mechanical homogenization for one minute using a sterile pestle. Hive debris samples were treated using the same protocol to maintain comparability across matrices. Homogenates were centrifuged at 10,000× *g* for 5 min at 4 °C to separate spores and particulate material from soluble components. The resulting pellet was resuspended in 1 mL of double-distilled water and stored at −20 °C until DNA extraction and molecular testing.

Preliminary microscopy was used to confirm the presence of *N. ceranae* spores before molecular testing. For this step, 10 µL of the homogenate was placed on a glass slide and examined under a compound light microscope (Leica DM500, Leica Microsystems, Wetzlar Germany) at 200× and 400× magnification. Microscopy provides initial visualization and morphological confirmation of *N. ceranae* spores, but it lacks the sensitivity of molecular assays for detecting low-level infections [26].

### 2.3. Molecular Diagnostics: qPCR and Digital PCR Methods

Quantitative PCR (qPCR) and droplet digital PCR (ddPCR) were employed for the detection and quantification of *N. ceranae* DNA. Both assays targeted the Hsp70 gene [38], which provides species-level discrimination within the genus.

#### 2.3.1. qPCR Procedure

The qPCR was performed with species-specific primers and a TaqMan probe:

- Forward 5′-GGGATTACAAGTGCTTAGAGTGATT-3′
- Reverse 5′-TGTCAAGCCCATAAGCAAGTG-3′
- Probe 5′-FAM-TGAGCCTACTGCGGC-TAMRA-3′.

Reactions (20 µL) contained 10 µL of Universal Master Mix II (Bio-Rad Laboratories, Hercules, CA, USA), plus 7 µL consisting of 0.5 µM of each primer, 0.25 µM probe, 2 µL of template DNA, and 1 µL of ExoIPC™ internal control (Applied Biosystems, Foster City, CA, USA) to monitor inhibition. Amplification was performed on a CFX96 Real-Time PCR System (Bio-Rad) with the following cycling conditions: 95 °C for 5 min, then 40 cycles of 95 °C for 15 s and 56 °C for 60 s. Fluorescence data were collected at the end of each extension phase and analyzed using CFX Manager software V3.1.

Each run included positive controls (DNA from a confirmed *N. ceranae* infection) and no-template controls (NTCs). Each sample was tested in duplicate, and replicates were accepted when Ct values differed by ≤0.5 cycles. Samples with fluorescence curves crossing the analytical threshold within 35 cycles (Ct ≤ 35) were considered positive. Ct values above 35 were classified as borderline, indicating low target DNA near the detection limit. Borderline samples were re-analyzed by ddPCR for confirmatory quantification. The validity of the ExoIPC signal in negative samples confirmed the absence of PCR inhibitors. The qPCR protocol is in agreement with the core recommendations of the MIQE guidelines [43], with particular attention to reporting transparency, reaction parameters, and validation criteria relevant to diagnostic applications.

#### 2.3.2. ddPCR Procedure

Droplet digital PCR (ddPCR) was used for absolute quantification and confirmation of borderline qPCR results. The same primers and probe were used to maintain consistency across platforms, with reaction conditions optimized for partitioned amplification in oil emulsion.

Each 20 µL reaction contained 10 µL of ddPCR Supermix for Probes (no dUTP) (Bio-Rad 1000 Alfred Nobel Drive Hercules, CA 94547 USA), 0.5 µL of each primer (20 µM stock, resulting in 0.5 µM final), 0.25 µL of probe (20 µM stock, resulting in 0.25 µM final), 2 µL of DNA template, and 6.75 µL of ddH_2_O.

Droplets (~20,000 per well) were generated using a QX200 Droplet Generator (Bio-Rad) with Droplet Generation Oil for Probes. Thermal cycling was performed in a C1000 Touch Thermal Cycler (Bio-Rad) under the following conditions: enzyme activation at 95 °C for 10 min; 40 cycles of 94 °C for 30 s and 55 °C for 60 s; enzyme deactivation at 98 °C for 10 min; hold at 4 °C.

After amplification, droplets were read with a QX200 Droplet Reader and analyzed using QXManager 2.0 software (Bio-Rad). Absolute quantification was computed by Poisson statistics as copies per µL of reaction [44]. The sample was analyzed in duplicate at different dilutions, showing consistent droplet counts and detection of positive droplets, according to digital MIQE guidelines [43]. NTCs and positive controls were included in each run to validate results. All assays were performed in duplicate to ensure reproducibility.

This combined qPCR/ddPCR approach allowed semi-quantitative screening followed by absolute quantification of infection load, providing a robust diagnostic framework for *N. ceranae* detection in bee and debris samples. Droplet were analyzed with the QX200 Droplet Reader and QXManager 2.0 software (Biorad).

### 2.4. Data Processing and Concordance Analysis

Data analysis was performed using IBM SPSS Statistics v26 (IBM Corp., Armonk, NY, USA) and GraphPad Prism v10.0 (GraphPad Software, San Diego, CA, USA). Descriptive statistics were calculated separately for bees and debris samples, and for each diagnostic platform (qPCR and ddPCR).

Agreement between qPCR and ddPCR results was assessed as the proportion of samples yielding identical qualitative outcomes (positive or negative). A secondary concordance rate was calculated after excluding qPCR results with Ct values > 35, which are considered near the detection limit. The ddPCR results were used as reference standards for sensitivity estimation, given their higher analytical precision and absolute quantification capability [44].

DNA copy numbers from ddPCR served as a proxy for spore load, enabling direct comparison between bees and debris samples. Copy number data (copies/µL) were log-transformed to improve normality prior to statistical testing. A one-way ANOVA was used to assess differences in infection intensity between diagnostic platforms and sample types. Post hoc pairwise comparisons were conducted using Tukey’s test when appropriate.

All qPCR and ddPCR assays were run in duplicate, and replicate means were used in subsequent analyses. Spore counts via hemocytometer were not performed; instead, ddPCR-based absolute quantification was used as the operational measure of infection intensity.

AI language tools were used to improve grammar and style; scientific interpretation remained solely the authors’ responsibility.

## 3. Results

### 3.1. Molecular Diagnostics and ddPCR Results

qPCR assays were conducted on a total number of 86 samples collected from three different apiaries, including bee bodies as well as hive debris. This qPCR analysis aimed to detect *N. ceranae* by targeting species-specific sequences in real time using a TaqMan probe-based approach. Out of the samples tested, a total number of 40 exhibited positive results, consisting of 36 samples from bee bodies as well as 4 samples from debris.

Positivity was determined according to the amplification plots and Ct threshold analysis described in the Methods, with internal controls confirming the absence of inhibition in negative samples. On this basis, qPCR defined the initial set of positive and negative samples, which then served as the reference for subsequent ddPCR analysis. ddPCR was applied to all samples except 10 debris samples that were qPCR-negative with Ct values above 30, indicating very low target DNA concentration. ddPCR confirmed all qPCR-positive samples and detected additional low-level positives in hive debris [44].

**Figure 1:**
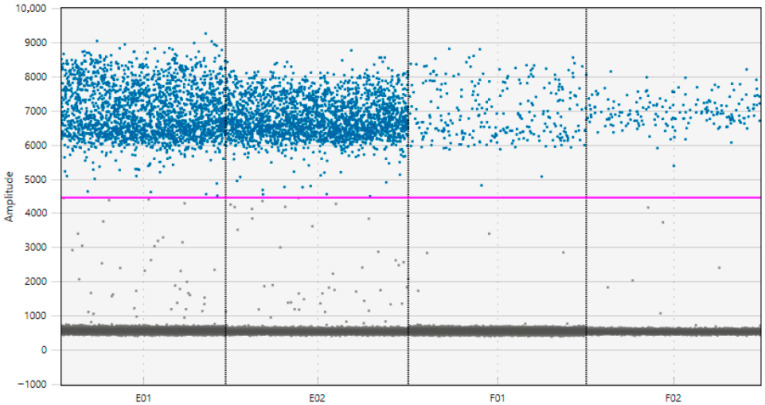
Droplet amplitude distribution plot from ddPCR analysis of Sample 61. The Figure shows the fluorescence amplitude of each droplet in wells E01 and E02 (1:10 dilution) and F01 and F02 (1:100 dilution), targeting *Nosema ceranae* DNA. Positive droplets (blue) cluster around high fluorescence values, clearly separated from negative droplets (gray) by the pink threshold line. The separation between signal and background demonstrates the specificity and sensitivity of the assay. This sample illustrates a typical result with a high parasitic load, as confirmed by the dense positive signal even after serial dilution.

For instance, sample in Figure 1 was selected as a representative case to illustrate in detail the utility of digital PCR (ddPCR) for *N. ceranae* DNA detection and quantification. The sample was analyzed in duplicate at various dilutions, showing consistent droplet counts and detection of positive droplets. In Figure 1, the results show the sensitivity of ddPCR in detecting low-copy DNA, pointing out its potential in distinguishing weak positives. Droplet generation is a crucial step in ddPCR. Higher concentrations (1:10) of template DNA, as in the first two wells, tend to lead to more detectable positive droplets. When the DNA is diluted more heavily, the total number of detectable positive droplets decreases, as seen in the F01 and F02 wells (1:100).

**Figure 2:**
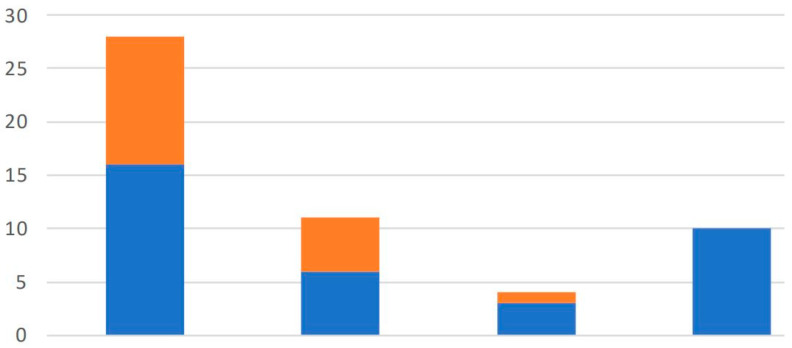
Classification of *N. ceranae*-positive samples detected by ddPCR, based on infection severity. Bars represent the number of samples assigned to each category: Weak, Intermediate, and Strong, according to parasite load thresholds defined in the Methods. The leftmost bar (“Traces”) shows the total number of positive detections. Color coding distinguishes the sample matrix: blue for adult bees; orange for hive debris.

The analysis of *N. ceranae* parasite loads using digital PCR (ddPCR) revealed significant variability among apiaries. For the qPCR assay, positive samples were divided into four distinct groups Traces (<5 copies/µL), Weak (5–100 copies/µL), Intermediate (100–1000 copies/µL), and Strong (>1000 copies/µL). This classification allowed a more precise estimation of infection intensity, clearly distinguishing between bees and debris samples. Of the 53 positive samples distinguished by ddPCR, the Traces group included 16 bees and 12 debris samples, and the Strong group included 10 bees with no related debris samples. Figure 2 shows the distribution within the above groups and the extensive difference in infection strength between bees and debris samples.

**Figure 3:**
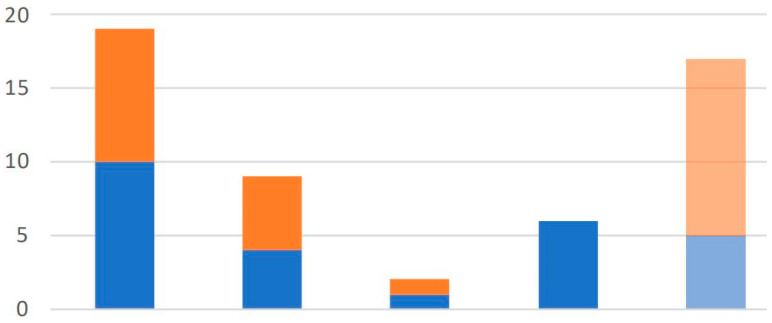
ddPCR-based classification of *Nosema ceranae* infection severity in samples from Apiary 1. Bars show the number of samples assigned to each category (from left): total positives (“Total”), Weak, Intermediate, Strong, and Negative. Blue bars indicate adult bee samples; orange bars indicate hive debris. The results illustrate how ddPCR distinguishes infection intensities and reveals additional positive detections in debris that are not captured in bee samples. The final bar group (“Negative”) includes samples where no *N. ceranae* DNA was detected by ddPCR. ‘Total’ bar is the sum of all the categories (‘Strong’, ‘Intermediate’, ‘Weak’, ‘Trace’) shown in the chart.

A subsequent analysis was performed on the parasite loads detected at Apiary 1 and Apiary 2. A total of 54 specimens were collected from Apiary 1, which included 27 bees and 27 debris samples. Many samples from this apiary tested positive for *N. ceranae*, with 21 bees and 15 debris samples giving positive diagnosis for the presence of infection. On the other hand, the negative results were limited to 5 bees and 12 debris samples. A more precise subclassification was conducted on the positive samples according to the parasite load observed, and 19 samples had Traces (10 bees and 9 debris), 9 had Weak (4 bees and 5 debris), 2 had Intermediate (1 bee and 1 debris), and 6 had Strong (all from bees). The parasite load in bees in Apiary 1 was measured at 1384.67 copies/µL compared to 17.56 copies/µL in debris. Figure 3 shows the results from Apiary 1 with a focus on the overwhelming number of positive samples and the distribution in the four categories designated by subclassification.

**Figure 4:**
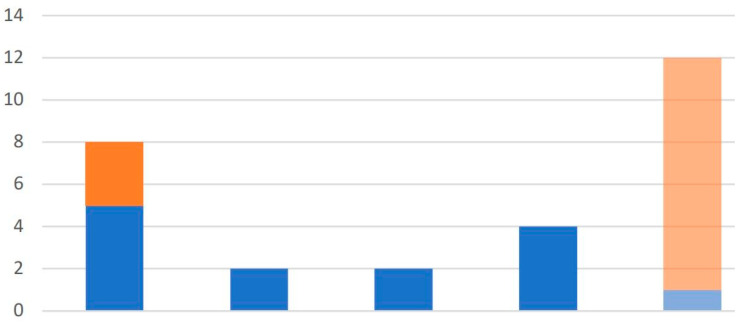
ddPCR-based classification of *Nosema ceranae* infection severity in samples from Apiary 2. Bars represent the number of samples in each category (from left): total positives (“Total”), Weak, Intermediate, Strong, and Negative. Blue bars indicate adult bee samples; orange bars indicate hive debris. In Apiary 2, most detections correspond to low- and moderate-intensity infections, with only a few Strong positives. The last column (“Negative”) includes all samples with no detectable parasite DNA. This figure illustrates how ddPCR allows nuanced detection across severity classes, and again confirms the utility of debris sampling in colony-level surveillance. ‘Total’ bar is the sum of all the categories (‘Strong’, ‘Intermediate’, ‘Weak’, ‘Trace’) shown in the chart.

In Apiary 2, a total of 28 samples were collected, comprising 14 bees and 14 debris. Most of the bee samples tested positive with 13 bees showing positivity and only 1 negative finding noted. Of the debris samples, 3 tested positive and 11 showed negative results. All the positive samples were thus classified in the same pattern as the samples from Apiary 1, where 8 samples were classified as Traces (consisting of 5 bees and 3 debris), 2 as Weak (2 bees and 0 debris), 2 as Intermediate (2 bees and 0 debris), and 4 as Strong (all from bees). The bees from Apiary 2 had on average 738.85 parasite loads/µL, in sharp contrast with the much smaller number from debris of 0.15 parasite loads/µL. Figure 4 shows the quantitative findings for Apiary 2 where the infection intensities in the bees were significantly more compared with those in debris.

### 3.2. Comparative Analysis of qPCR and ddPCR Performances

Apiary 1 exhibited higher parasite loads in both debris and bee samples, indicating greater overall infection than Apiary 2. Differences observed during sampling may reflect local environmental conditions or management practices; our cross-sectional design does not allow causal inference. Apiary 2 had fewer positive debris samples, and smaller average parasite counts in bees and debris samples. That is likely the result of local conditions or the health status of the bee colonies. These findings highlight the influence of local conditions on *N. ceranae* transmission and underscore the utility of ddPCR in quantifying infection levels under varied field conditions [44].

Figure 2, Figure 3 and Figure 4 indicate a distinct discrimination between negative and positive samples according to the fluorescence signals in ddPCR. These results confirm the reliability of ddPCR-based molecular quantification.

Side-by-side comparison of ddPCR and qPCR results revealed substantial agreement. Overall, ddPCR identified a total of 35 positive samples, a number slightly lower than the 36 detected by qPCR. However, ddPCR demonstrated its superior sensitivity specifically in debris samples, detecting 18 positive samples compared to only 4 detected by qPCR. This yielded an agreement rate of 95.24%, which increased to 96.88% when weak positives (Ct > 35) were excluded. That indicates both methods performed well at accurately identifying moderate-to-high infection.

In debris samples, ddPCR showed superior sensitivity. It detected 18 positive samples, whereas 4 were detected by qPCR. This demonstrates ddPCR’s ability to detect low-intensity infections undetected by qPCR.

Pearson analysis demonstrated a negative correlation between the qPCR Ct values and the ddPCR copy numbers in the positive bee samples (r = −0.75, *p* < 0.001). The finding verifies the fact that both methods yield comparable outcomes when conducted on the samples harvested from the fields. Both qPCR and ddPCR effectively detect *N. ceranae*, but ddPCR offers greater sensitivity for low-level or borderline infections. That serves in disease pattern analysis and observation on the health of colonies.

### 3.3. Statistical Analysis of Diagnostic Results

We statistically compared qPCR and ddPCR results to assess diagnostic agreement, infection quantification, and variability between apiaries in *N. ceranae* detection. Data were derived from 86 samples originating from three Northern Italian apiaries, comprising both bee bodies and hive debris, as reported above.

#### 3.3.1. Correlation Between qPCR Ct Values and ddPCR Copy Numbers

The relationship between the semi-quantitative cycle threshold (Ct) values obtained by qPCR and the absolute copy number measurements from ddPCR was examined using Pearson’s correlation coefficient. The full dataset showed a weak negative correlation (r = −0.13), likely due to negative and low-load samples with noisy amplification. When the analysis was restricted to positive samples, the correlation became strongly negative (r = −0.71) and increased further when samples with Ct > 35 were excluded (r = −0.75). This confirms the expected inverse relationship between Ct and target copy number, demonstrating that qPCR is a reliable proxy for infection intensity within its dynamic range but loses quantitative precision at high Ct values near the detection threshold.

#### 3.3.2. Diagnostic Concordance Between Assays

Concordance between the two molecular assays was assessed by comparing the presence or absence of amplification in paired qPCR and ddPCR results. Among bee samples, 34 of 35 ddPCR-positive cases were also qPCR-positive; one was positive only by ddPCR. The overall diagnostic concordance was 95%, increasing to 97% after exclusion of high-Ct cases (Ct > 35). Cohen’s κ coefficient for inter-method agreement was 0.93 (95% CI: 0.86–1.00), indicating almost perfect agreement. These results confirm that both assays identify infected colonies reliably, though ddPCR offers superior sensitivity for low-load or borderline cases—especially in debris.

#### 3.3.3. Distribution of Infection Loads

Quantitative results from ddPCR were used to characterize infection intensity. Based on ddPCR copy number per µL, positive samples were categorized as: trace (<5), weak (5–100), intermediate (100–1000), and strong (>1000). The majority of positive samples fell within the lower two categories, confirming that subclinical or early-stage infections predominated in this cohort. In bees, ddPCR detected 16 traces, 6 weak, 3 intermediate, and 10 strong infections. In debris, 12 were traces, 5 weak, 1 intermediate, and none strong. These findings emphasize that debris acted as a reservoir for low-level contamination rather than active infection.

#### 3.3.4. Between-Apiary Comparison

A non-parametric Kruskal–Wallis test was performed to compare ddPCR-measured parasite loads between apiaries, followed by pairwise post hoc comparisons (Dunn’s test). Apiary 1 exhibited the highest overall load, with mean and ±SD: copy numbers of 1384.67 ± 312.4 copies/µL in bees and 17.56 ± 6.2 copies/µL in debris. Apiary 2 showed significantly lower loads (bees: 738.85 ± 128.7 copies/µL; debris: 0.15 ± 0.08 copies/µL; *p* < 0.05), while data from Apiary 3 were excluded due to insufficient sample size. Median infection levels differed significantly between Apiaries 1 and 2 (H = 6.21, *p* = 0.013), confirming infection heterogeneity.

#### 3.3.5. Interpretation

Overall, these analyses demonstrate strong diagnostic consistency between qPCR and ddPCR, with ddPCR providing enhanced sensitivity and quantitative precision, especially for low-intensity or environmentally derived samples. The inverse correlation between Ct and copy number supports the biological validity of both assays, while the observed between-apiary variation highlights the heterogeneous distribution of *N. ceranae* infections within the same geographic region. Combining qPCR for high-throughput screening with ddPCR for confirmatory quantification offers a statistically robust framework for disease monitoring.

## 4. Discussion

### 4.1. Colony-Level Impacts

High *N. ceranae* loads impair colony strength and increase vulnerability to pathogens [45,46,47]. Previous studies reported that honey production decreases by up to 40% when spore loads exceed 10^6^ per bee, accompanied by a marked decrease in the number of active foragers [48,49]. The consequences of these relationships are represented in Table 2, which illustrates the impacts of *N. ceranae* infections on colony productivity.

The high infection intensities measured, particularly by ddPCR in debris samples, are consistent with productivity losses reported in previous studies [50]. This confirms the importance of molecular diagnostics for the early identification of colony-level impacts [51].

In addition, *N. ceranae* infection increases susceptibility to secondary pathogens, notably *Deformed Wing Virus* (*DWV*), further reducing colony vigor and contributing to 30–50% declines in population growth compared to minimally infected colonies [52]. Such effects highlight how parasitic burden and viral co-infections act synergistically to weaken hives and accelerate colony collapse.

Since all samples in our study were collected during spring and summer, the elevated infection levels we observed are consistent with literature showing that *N. ceranae* proliferates most aggressively under warm and humid conditions [42]. This seasonal correspondence underscores the importance of time-specific surveillance and management during high-risk periods. However, this study was limited to one season and few location; future research should cover more diverse apiaries.

These infestations have substantial economic consequences, particularly for commercial beekeeping operations. Reduced honey production lowers the profitability of both small-scale and industrial apiaries [52]. Routine debris removal, and improved hive sanitation are among the management measures that can mitigate infection pressure.

The colony-level impacts described above ultimately stem from the biological cycle and transmission dynamics of *N. ceranae*. Understanding how the parasite persists and spreads within the hive environment is essential for interpreting diagnostic results and developing effective control measures. In this context, molecular evidence from the present study—particularly the detection of *N. ceranae* DNA in debris by ddPCR—provides direct insight into the environmental stages of the infection cycle. These findings bridge laboratory diagnostics with the ecological processes that sustain colony-level infections [53].

### 4.2. Integrating Lifecycle Dynamics with Diagnostic Strategies

The Nosema ceranae lifecycle begins when adult bees ingest environmentally stable spores. Spores germinate in the midgut, multiply within epithelial cells, mature, and then shed via feces, accumulating in the hive’s wax and floor debris. This environmental contamination sustains infectious pressure within colonies [53].

The detection of *N. ceranae* DNA in debris using ddPCR confirms this persistent environmental phase of the cycle. ddPCR serves as an early-warning tool, quantifying low-level environmental contamination before symptoms manifest in foraging bees [54,55].

The spore resilience in debris highlights the need for sanitation to interrupt transmission cycles [56]. Understanding the lifecycle helps beekeepers identify effective intervention points, such as pre-season cleaning and post-winter surveillance, when spore accumulation is likely to peak [57].

The qPCR and ddPCR techniques fit into the lifecycle framework as first-line and confirmatory strategies, respectively, enabling comprehensive monitoring across both biological (bees) and environmental (debris) compartments.

### 4.3. Comparative Performance of qPCR and ddPCR in Bees and Debris

Our study exclusively utilized positive field samples, rather than artificially ‘spiked’ ones, acknowledging that real environmental matrices like debris are highly heterogeneous and harbor inhibitors that can obscure the true parasite load.

In the diagnostic comparison, qPCR and ddPCR exhibited high concordance in bee samples, confirming that qPCR is a robust method for detecting moderate-to-high infection loads. However, its reliability wanes near the detection threshold (high Ct) due to amplification variability and inhibitor effects on the fluorescence signal. This is a critical limitation, as borderline results can lead to the underestimation of prevalence [58].

The ddPCR system overcomes these issues; its architecture, which partitions the reaction into thousands of droplets, effectively minimizes inhibitor influence and significantly enhances the likelihood of detecting low-copy targets, a technical advantage reported in other veterinary systems [59,60].

This superiority was most pronounced in the complex debris matrix, where ddPCR detected 18 positive samples compared to only 4 identified by qPCR. As debris accumulates material over time—providing a broader temporal window than the single snapshot offered by adult bees—ddPCR’s heightened sensitivity allows it to detect early-stage or subclinical infections not yet evident in the adult bee population, establishing its value as a sensitive, non-invasive diagnostic approach [61]. Furthermore, the parasite load quantification provided by both methods enables the necessary diagnostic stratification of colony infection intensity, crucial for evidence-based veterinary intervention and monitoring [62].

In conclusion, our results advocate for a combined diagnostic framework where ddPCR provides the absolute quantitative precision—without the need for a standard curve—ideal for research and confirmation. This combined approach allows bee health monitoring systems to balance efficiency with precision. Nevertheless, the routine practical implementation of ddPCR is currently constrained by the significantly higher cost of equipment and consumables, and its droplet-based format complicates downstream applications like sequencing. Therefore, while ddPCR demonstrates superior performance in detection limits, the qPCR remains the more accessible and operationally flexible method for routine surveillance programs. To our knowledge, this is the first study to employ ddPCR for the detection and quantification of *N. ceranae* in both bee and debris samples.

## 5. Conclusions

This study presents the first integrated application of droplet digital PCR (ddPCR) for detecting and quantifying *N. ceranae* in both bee and hive debris samples. ddPCR complements qPCR by offering higher sensitivity and accuracy. Combining both techniques enables early detection and reliable monitoring of *N. ceranae* infections. While qPCR remains a practical first-line tool for large-scale surveillance, ddPCR provides precise quantification suited for confirmatory testing and epidemiological studies.

Integrating these molecular tools into a complementary framework allows bee health monitoring to combine operational feasibility with diagnostic precision. This dual approach improves prevalence estimates and supports evidence-based colony health management. The framework established here lays the groundwork for standardized, sensitive, and scalable molecular surveillance of *N. ceranae*, advancing proactive and sustainable apicultural disease control. This diagnostic integration may enhance national bee health surveillance programs.

## Figures and Tables

**Table 1:** Sampling sessions with Apiary ID, municipality and location, number of colonies sampled for each matrix (honey bee and hive debris).

Apiary ID	Municipality	No. of Sampled Colonies (Honey Bee)	No. of Sampled Colonies (Hive Debris)
1	Cocconato d’Asti (AT)	27	27
2	Castelnuovo Don Bosco (AT)	14	14
3	Asti (AT)	2	2

**Table 2:** Impacts of *N. ceranae* Infections on Colony Productivity.

Indicators	Impact	Quantitative Data
Foraging Efficiency	Decreased active foragers and resource intake	~30% fewer active foragers in infected colonies [46,47]
Secondary Infections	Increased susceptibility to pathogens	High correlation with DWV and other Viruses [45,46,47]
Population Growth Rates	Reduced worker replacement and brood rearing	30–50% lower growth rates in heavily infected colonies [46,47]

## Data Availability

The original contributions presented in this study are included in the article. Further inquiries can be directed to the corresponding author.

## Abbreviations

The following abbreviations are used in this manuscript:

qPCR	Real-time PCR
ddPCR	Droplet Digital PCR
NTCs	no-template controls
Ct	threshold cycle
